# Higher erythrocytes in cerebrospinal fluid on the first and seventh postoperative day: Associated with poor outcome in aneurysmal subarachnoid hemorrhage patients

**DOI:** 10.1097/MD.0000000000040027

**Published:** 2024-10-11

**Authors:** Jie Min, Yongfeng Zhao, Xian Wang, Jian Zhao

**Affiliations:** a Neurointensive Care Unit, The First Affiliated Hospital of Yangtze University, Jingzhou, China; b Department of Hematology, The First Affiliated Hospital of Yangtze University, Jingzhou, China; c Department of Pharmacy, The First Affiliated Hospital of Yangtze University, Jingzhou, China.

**Keywords:** aneurysmal subarachnoid hemorrhage, cerebrospinal fluid, erythrocytes, outcome

## Abstract

Aneurysmal subarachnoid hemorrhage is an acute cerebrovascular disease with high disability and mortality. We intended to explore the association between levels of erythrocytes in cerebrospinal fluid at different times of hemorrhage and the outcome of patients. One retrospective study including 216 patients with aneurysmal subarachnoid hemorrhage undergoing surgeries in the First Affiliated Hospital of Yangtze University from January 2020 to July 2023 was carried. The univariable analysis and multivariable logistic regression analysis were used for factors associated with poor outcome. The level of erythrocytes in cerebrospinal fluid on the 1st postoperative day in patients with poor outcome was 311 × 10^3^/µL, significantly higher than patients with good outcome (108 × 10^3^/µL), *P* < .001. The level of erythrocytes in cerebrospinal fluid on 7th postoperative day was 86.5 × 10^3^/µL, also significantly higher than patients with good outcome (26.0 × 10^3^/µL). The multivariable logistic regression analysis results showed that erythrocytes in cerebrospinal fluid on the 1st postoperative day (≥177 × 10^3^/µL) and on the 7th postoperative day (≥53.5 × 10^3^/µL) were possibly associated with poor outcome in aneurysmal subarachnoid hemorrhage patients. Treatment with tranexamic acid and continuous lumbar drainage did not result in a decrease of erythrocytes in cerebrospinal fluid. Higher erythrocytes in cerebrospinal fluid on the 1st and 7th postoperative days were associated with poor outcome in aneurysmal subarachnoid hemorrhage patients.

## 1. Introduction

Aneurysmal subarachnoid hemorrhage (aSAH) is an acute cerebrovascular disease that severely damages the central nervous system and has pathological effects on multiple organs.^[1]^ Recurrent bleeding, advanced age, and higher Hunt–Hess grade was possibly associated with poor outcome in these patients.^[2,3]^ So far, craniotomy clipping and endovascular embolization are the main methods for preventing and treating aSAH, improving life quality of patients.^[4]^ Nevertheless, cerebral vasospasm and delayed cerebral ischemia were serious complications in patients with aSAH.^[5]^

Cerebral vasospasm occurred in approximately 70% to 90% patients with aSAH, significantly reducing blood flow in brain. Severe vasospasm could devastate outcomes of patients.^[5,6]^ The blood in subarachnoid space was considered 1 of the important factors associated with cerebral vasospasm. Hemoglobin released from dissolved erythrocytes on the second to fourth day after hemorrhage played an important role in inducing vasospasm.^[7]^ Hemoglobin in cerebrospinal fluid not only induced vasoconstriction, but also was associated with secondary brain injury after subarachnoid hemorrhage. Hemoglobin in cerebrospinal fluid could serve as a marker for secondary brain injury.^[8]^ However, there was limited study on the association between erythrocytes in cerebrospinal fluid and the outcome of aSAH patients. After occurrence of subarachnoid hemorrhage, the blood in subarachnoid space is dynamic. So the main purpose of our study is to explore the association between levels of erythrocytes in cerebrospinal fluid at different times of hemorrhage and the outcome of patients.

## 2. Methods

There were 216 patients with aSAH undergoing surgeries in the First Affiliated Hospital of Yangtze University from January 2020 to July 2023 included in this retrospective study. The inclusion criteria were age ≥ 18 years; patients with subarachnoid hemorrhage caused by aneurysm rupture confirmed by Computed Tomographic Angiography and/or Digital Subtraction Angiography (DSA) examinations; and patients receiving craniotomy clipping or intravascular embolization surgeries after hemorrhage. The exclusion criteria were: patients with subarachnoid hemorrhage caused by arteriovenous malformations, trauma, or other reasons, without aneurysm found by Computed Tomographic Angiography and/or Digital Subtraction Angiography; patients with severe hematological disorders before onset; patients with the modified Rankin scale (mRS) score ≥ 3 before onset; patients with an untreated meningitis before onset; and patients unable to complete follow-up. This retrospective study was reviewed and approved by the Ethics Committee of the First Affiliated Hospital of Yangtze University. The patients in this study were all treated by the aSAH professional team in the hospital, including neurosurgeons, clinical pharmacists, and nursing staff. The management plan included early craniotomy clipping or intravascular embolization surgeries, neurointensive care, and drug therapy.

Baseline characteristics included age, gender, concomitant intraventricular hemorrhage, Glasgow coma scale (GCS) score, World Federation of Neurosurgical Societies (WFNS) grade, Fisher grade, parameters of aneurysm (maximum diameter and neck diameter), surgical mode, and surgical time. Pressure of cerebrospinal fluid, erythrocytes or red blood cells, leukocytes or white blood cells in cerebrospinal fluid on the 1st, 3rd, and 7th postoperative days were recorded. The clinical outcome was determined by 90-d mRS. The patient with mRS ≤ 2 was defined as having a good outcome, while with mRS ≥ 3 was defined as having a poor outcome. The above data were all extracted from the hospital information system.

SPSS 23.0 (IBM SPSS Inc., Chicago, USA) was used for data analysis. The qualitative variables were compared by Pearson chi-square test, Continuity correction or Fisher exact test. If the quantitative variables met the normal distribution and variances were homogeneous, independent-sample *t*-test was used for analysis. If the quantitative variables met the normal distribution but variances were not homogeneous, corrected *t*-test was used. If the normal distribution could not be satisfied, Mann–Whitney *U* test was used for analysis. The multivariable logistic regression model was used for factors associated with poor outcome. Covariates that had a *P*-value <0.05 in the univariable analysis were added to the multivariable logistic regression model. The receiver operating characteristic curve (ROC) was used for the Youden index and cutoff value analysis. All *P*-values were 2-sided and the statistical significance was set at *P* < .05.

## 3. Results

### 3.1. Baseline characteristics

There were 84 males and 132 females among the included 216 aSAH patients in the final analysis. The average age was 60 years old. There were 95 patients combined with intraventricular hemorrhage, and 38 patients with GCS score < 11. A total of 45 patients were WFNS grades IV and V, and 75 patients were Fisher grades III and IV. There were 173 cases with intravascular embolization surgeries and 43 cases with craniotomy clipping surgeries. The median surgical duration was 170 minutes. The median maximum diameter of aneurysm was 4.90mm, and the neck diameter was 3.45mm. There were a total of 110 cases receiving antifibrinolytic agent (Table 1).

**Table 1:** Characteristics of all included aneurysmal subarachnoid hemorrhage patients.

	Total (n = 216)	Good outcome (n = 159)	Poor outcome (n = 57)	*P*
Gender [n (%)]				
Male	84 (38.9%)	57 (35.8%)	27 (47.4%)	0.126
Female	132 (61.1%)	102 (64.2%)	30 (52.6%)	
Age (yr)	60 ± 9	59 ± 10	62 ± 9	0.021
With intraventricular hemorrhage [n (%)]	95 (44.0%)	56 (35.2%)	39 (68.4%)	<0.001
Glasgow coma scale [n (%)]				
≥11	178 (82.4%)	146 (91.8%)	32 (56.1%)	<0.001
<11	38 (17.6%)	13 (8.2%)	25 (43.9%)	
WFNS grade [n (%)]				
I to III	171 (79.2%)	141 (88.7%)	30 (52.6%)	<0.001
IV and V	45 (20.8%)	18 (11.3%)	27 (47.4%)	
Fisher grade [n (%)]				
I and II	141 (65.3%)	124 (78.0%)	17 (29.8%)	<0.001
III and IV	75 (34.7%)	35 (22.0%)	40 (70.2%)	
Surgical mode [n (%)]				
Embolization	173 (80.1%)	136 (85.5%)	37 (64.9%)	0.001
Clipping	43 (19.9%)	23 (14.5%)	20 (35.1%)	
Surgical duration	170 ± 88	160 ± 80	190 ± 90	0.013
Diameter of aneurysm neck (mm)	3.45 ± 1.52	3.48 ± 1.48	3.25 ± 2.18	0.850
Maximum diameter (mm)	4.90 ± 2.96	4.54 ± 2.64	5.24 ± 3.63	0.199
Antifibrinolytic agent				
Yes	110 (50.9%)	82 (51.6%)	28 (49.1%)	0.751
No	106 (49.1%)	77 (48.4%)	29 (50.9%)	

The average age of patients with poor outcome was 62 years old, older than patients with good outcome (59 years), *P* = .021. There were 39 (68.4%) cases with intraventricular hemorrhage in patients with poor outcome, significantly more than patients with good outcome (35.2%), *P* < .001. The proportions of cases with GCS score < 11 (43.9% vs 8.2%), WFNS grade IV and V (47.4% vs 11.3%), and Fisher grade III-IV (70.2% vs 22.0%) in patients with poor outcome were all significantly higher than patients with good outcome, *P* < .001. There were 136 (85.5%) cases undergoing intravascular embolization surgeries in patients with good outcome, more than patients with poor outcome (64.9%), *P* = .001. The surgical duration in patients with poor outcome was 190 ± 90 minutes, longer than patients with good outcome (160 ± 80 minutes), *P* = .013. There were no significant differences in gender, maximum diameter, neck diameter of aneurysm, proportion of treatment with antifibrinolytic agent between patients with poor outcome and good outcome, *P* > .05 (Table 1).

### 3.2. Factors associated with outcome

The cerebrospinal fluid pressure in all included patients were 285 ± 100, 200 ± 113, and 193 ± 69 mmH_2_O, respectively, on the 1st, 3rd, and 7th postoperative days. On these days, the level of erythrocytes in cerebrospinal fluid were 131 ± 253 × 10^3^/µL, 65.5 ± 103.9 × 10^3^/µL, and 34.5 ± 77.3 × 10^3^/µL, respectively. The level of leukocytes were 0.139 ± 0.319 × 10^3^/µL, 0.086 ± 0.240 × 10^3^/µL, and 0.206 ± 0.072 × 10^3^/µL, respectively (Table 2).

**Table 2:** Differences of cerebrospinal fluid measurement results in patients with different outcomes.

	Total	Good outcome	Poor outcome	*P*
The 1st postoperative day				
Erythrocytes (×10^3^/µL)	131 ± 253	108 ± 200	311 ± 221	<.001
Leukocytes (×10^3^/µL)	0.139 ± 0.319	0.114 ± 0.239	0.400 ± 0.576	<.001
Pressure (mmH_2_O)	285 ± 100	280 ± 100	300 ± 100	.291
The 3rd postoperative day				
Erythrocytes (×10^3^/µL)	65.5 ± 103.9	60.5 ± 93.5	126.5 ± 261.0	.078
Leukocytes (×10^3^/µL)	0.086 ± 0.240	0.070 ± 0.220	0.100 ± 0.416	.075
Pressure (mmH_2_O)	200 ± 113	200 ± 72	231 ± 65	.061
The 7th postoperative day				
Erythrocytes (×10^3^/µL)	34.5 ± 77.3	26.0 ± 35.8	86.5 ± 202.8	<.001
Leukocytes (×10^3^/µL)	0.206 ± 0.072	0.133 ± 0.348	0.451 ± 0.866	.001
Pressure (mmH_2_O)	193 ± 69	193 ± 75	195 ± 60	.912

**Figure 1.**
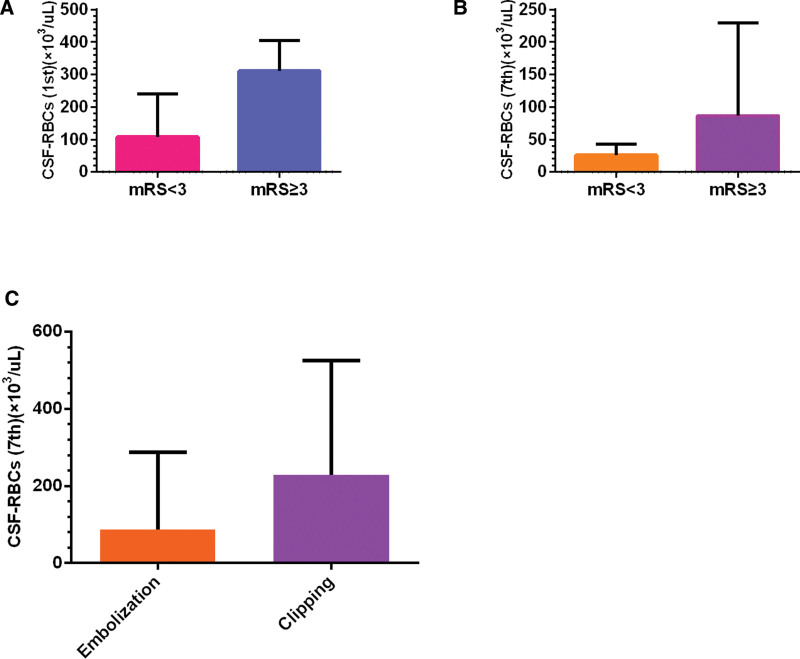
Differences of erythrocytes in cerebrospinal fluid on the 1st and 7th postoperative days. The level of erythrocytes in cerebrospinal fluid on the 1st postoperative day in patients with poor outcome was significantly higher than patients with good outcome, *P* < .001 (A). The level of erythrocytes in cerebrospinal fluid on the 7th postoperative day was also significantly higher than patients with good outcome, *P* < .001(B). The level of erythrocytes in cerebrospinal fluid on the 7th postoperative day in patients with clipping was significantly higher than patients with embolization, *P* = .019 (C).

The level of erythrocytes in cerebrospinal fluid on the 1st postoperative day in patients with poor outcome was 311 ± 221 × 10^3^/µL, significantly higher than patients with good outcome (108 ± 200 × 10^3^/µL), *P* < .001 (Fig. 1A). The level of erythrocytes in cerebrospinal fluid on the 7th postoperative day was 86.5 ± 202.8 × 10^3^/µL, also significantly higher than patients with good outcome (26.0 ± 35.8 × 10^3^/µL), *P* < .001 (Fig. 1B). The level of leukocytes in cerebrospinal fluid on the 1st postoperative day in patients with poor outcome was 0.400 ± 0.576 × 10^3^/µL, significantly higher than patients with good outcome (0.114 ± 0.239 × 10^3^/µL), *P* < .001. The level of leukocytes in cerebrospinal fluid on the 7th postoperative day was 0.451 ± 0.866 × 10^3^/µL, also higher than patients with good outcome (0.133 ± 0.348 × 10^3^/µL), *P* = .001 (Table 2).

**Figure 2.**
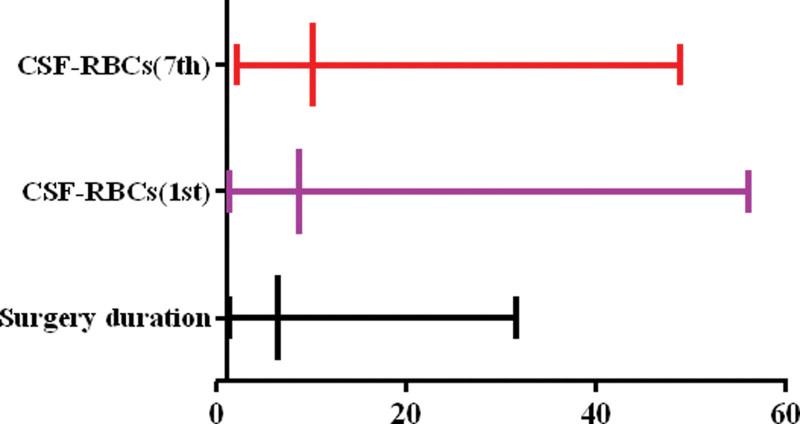
The multivariable logistic regression model of factors associated with poor outcome. The results of multivariable logistic regression analysis showed that surgical duration, erythrocytes in cerebrospinal fluid on the 1st postoperative day, and erythrocytes in cerebrospinal fluid on the 7th postoperative day were possibly associated with poor outcome in aSAH patients.

The results of multivariable logistic regression analysis showed that surgical duration (≥168.5 minutes) (OR = 6.425, 95% CI: 1.305–31.634, *P* = .022), erythrocytes in cerebrospinal fluid on the 1st postoperative day (≥177 × 10^3^/µL) (OR = 8.751, 95% CI: 1.363–56.181, *P* = .022), and erythrocytes in cerebrospinal fluid on the 7th postoperative day (≥53.5 × 10^3^/µL) (OR = 10.173, 95% CI: 2.115–48.925, *P* = .004) were possibly associated with poor outcome in aSAH patients (Fig. 2).

**Figure 3.**
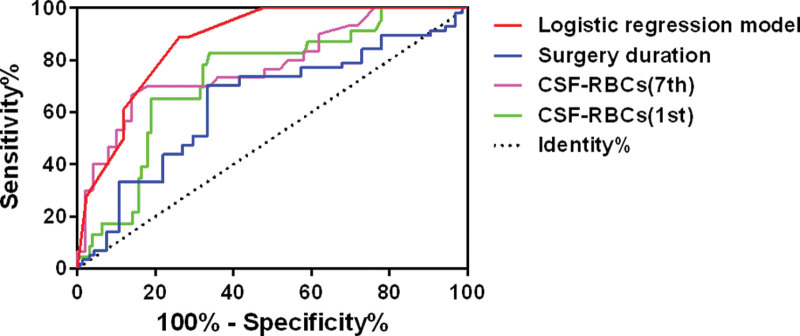
The receiver operating characteristic curves of different factors and the multivariable logistic regression model. The sensitivity and specificity of surgical duration for predicting outcome were 73.7% and 55.3%, respectively. The sensitivity and specificity of erythrocytes in cerebrospinal fluid on the 1st postoperative day were 82.6% and 66.1%, respectively. The sensitivity and specificity of erythrocytes in cerebrospinal fluid on the 7th postoperative day were 66.7% and 86.0%, respectively. The sensitivity and specificity of multivariable logistic regression model were 50% and 88.1%, respectively.

The cutoff value of surgical duration was 168.5 minutes (sensitivity: 73.7%, specificity: 55.3%, AUC: 0.610, *P* = .014). The cutoff values of erythrocytes in cerebrospinal fluid on the 1st and 7th postoperative days were 177 × 10^3^/µL (sensitivity: 82.6%, specificity: 66.1%, AUC: 0.732, *P* < .001) and 53.5 × 10^3^/µL (sensitivity: 66.7%, specificity: 86.0%, AUC: 0.779, *P* < .001), respectively. The positive predictive value of multivariable logistic regression model for predicting outcome in aSAH patients was 64.3%, and the negative predictive value was 80.4%. The sensitivity and specificity were 50% and 88.1%, respectively (Fig. 3).

We compared the effects of 2 different cerebrospinal fluid release methods on the levels of erythrocytes, leukocytes, and pressure in cerebrospinal fluid on the 1st and 7th postoperative days. On the 1st postoperative day, the level of erythrocytes in cerebrospinal fluid in patients with lumbar puncture was 104.1 ± 200 × 10^3^/µL, significantly lower than patients with lumbar cistern drainage (405 ± 858.5 × 10^3^/µL), *P* < .001. The level of leukocytes in cerebrospinal fluid in patients with lumbar puncture was 0.119 ± 0.218 × 10^3^/µL, significantly lower than patients with lumbar cistern drainage (0.614 ± 1.058 × 10^3^/µL), *P* < .001. On the 7th postoperative day, the levels of erythrocytes in cerebrospinal fluid in patients with lumbar puncture and lumbar cistern drainage showed no significant differences (33 ± 63 × 10^3^/µL vs 35 ± 138 × 10^3^/µL), *P* = .313. The level of leukocytes in cerebrospinal fluid in patients with lumbar puncture was 0.133 ± 0.452 × 10^3^/µL, significantly lower than patients with lumbar cistern drainage (0.434 ± 0.742 × 10^3^/µL), *P* = .010 (Table 3).

**Table 3:** Differences of cerebrospinal fluid measurement results in patients with different treatment methods.

	Lumbar puncture	Lumbar cistern drainage	*P*
The 1st postoperative day			
Erythrocytes (×10^3^/µL)	104.1 ± 200	405 ± 858.5	<.001
Leukocytes (×10^3^/µL)	0.119 ± 0.218	0.614 ± 1.058	<.001
Pressure (mmH_2_O)	280 ± 100	310 ± 163	.774
The 7th postoperative day			
Erythrocytes (×10^3^/µL)	33 ± 63	35 ± 138	.313
Leukocytes (×10^3^/µL)	0.133 ± 0.452	0.434 ± 0.742	.010
Pressure (mmH_2_O)	170 ± 115	200 ± 100	.118

	Embolization	Clipping	*P*
The 1st postoperative day			
Erythrocytes (×10^3^/µL)	126.5 ± 249.5	224.0 ± 279.8	.178
Leukocytes (×10^3^/µL)	0.139 ± 0.303	0.140 ± 0.364	.836
Pressure (mmH_2_O)	295 ± 100	220 ± 90	.042
The 7th postoperative day			
Erythrocytes (×10^3^/µL)	33 ± 72	61 ± 367	.019
Leukocytes (×10^3^/µL)	0.238 ± 0.433	0.267 ± 0.753	.365
Pressure (mmH_2_O)	193 ± 71	197 ± 53	.882

	Antifibrinolytic agent	Without antifibrinolytic agent	*P*
The 1st postoperative day			
Erythrocytes (×10^3^/µL)	152 ± 279	127 ± 195	.544
Leukocytes (×10^3^/µL)	0.150 ± 0.374	0.123 ± 0.290	.277
Pressure (mmH_2_O)	270 ± 115	300 ± 95	.842
The 7th postoperative day			
Erythrocytes (×10^3^/µL)	31.5 ± 81.3	41 ± 76.3	.726
Leukocytes (×10^3^/µL)	0.251 ± 0.420	0.201 ± 0.712	.730
Pressure (mmH_2_O)	194 ± 77	193 ± 59	.948

We compared the effects of 2 different surgical modes on the levels of erythrocytes, leukocytes, and pressure in cerebrospinal fluid on the 1st and 7th postoperative days. On the 7th postoperative day, the level of erythrocytes in cerebrospinal fluid in patients with clipping surgery was 61 ± 367 × 10^3^/µL, significantly higher than patients with embolization surgery (33 ± 72 × 10^3^/µL), *P* = .019 (Fig. 1C). In addition, the pressure of cerebrospinal fluid on the 1st postoperative day in patients with embolization surgery was 295 ± 100mmH_2_O, significantly higher than patients with clipping surgery (220 ± 90), *P* = .042. There was no significant difference in the levels of erythrocytes in cerebrospinal fluid on the 1st postoperative day between patients with 2 different surgical modes, *P* = .178 (Table 3).

We also explored the effects of antifibrinolytic agent on the levels of erythrocytes, leukocytes, and pressure in cerebrospinal fluid on the 1st and 7th postoperative days. There were no significant differences in the levels of erythrocytes in cerebrospinal fluid on the 1st (152 ± 279 vs 127 ± 195 × 10^3^/µL, *P* = .544) and 7th (31.5 ± 81.3 vs 41 ± 76.3 × 10^3^/µL, *P* = .726) postoperative days between patients with antifibrinolytic agent and without antifibrinolytic agent. There were also no significant differences in the levels of leukocytes and pressure in cerebrospinal fluid on the 1st and 7th postoperative days between patients with antifibrinolytic agent and without antifibrinolytic agent (Table 3).

## 4. Discussion

After subarachnoid hemorrhage, erythrocytes could be detected in the patient’s cerebrospinal fluid. The substances in cerebrospinal fluid that could lead to delayed brain injury or cerebral ischemia after subarachnoid hemorrhage included hemoglobin, oxidation products of iron and bilirubin, and other compounds during the degradation process of erythrocytes.^[9–12]^ Nozaki K et al tested the spasmodic activity of various blood components in dogs, erythrocytes were necessary for late and prolonged arterial stenosis after experimental subarachnoid hemorrhage.^[13]^ Compared with patients without delayed brain injury, an increase of erythrocytes in cerebrospinal fluid in patients with delayed cerebral ischemia was confirmed.^[14–16]^ The hemoglobin released by erythrocytes could induce cerebral vasospasm, possibly further affect the clinical outcome of patients. Cerebral vasospasm visible on imaging could be seen in 70% aSAH patients, while cerebral vasospasm visible clinically occurred in 20% to 30% aSAH patients.^[17]^ Cerebral vasospasm caused by basal pool blood was considered the main cause of delayed cerebral ischemia.^[18]^ There were 51.5% patients experiencing delayed cerebral ischemia.^[19]^ Our study confirmed that the counts of erythrocytes in cerebrospinal fluid varied on different postoperative days. The counts of erythrocytes in cerebrospinal fluid on the 1st and 7th postoperative days among patients with poor outcome were significantly higher than patients with good outcome. The results of multivariable analysis showed that counts of erythrocytes ≥ 177 × 10^3^/µL on the 1st postoperative day and ≥ 53.5 × 10^3^/µL on the 7th postoperative day were possibly associated with poor outcome. No associations of cerebrospinal fluid pressure and leukocytes in cerebrospinal fluid with outcome of patients were confirmed.

Craniotomy clipping and intravascular embolization were 2 important methods to treat aneurysmal subarachnoid hemorrhage. Consistent with the result of univariate analysis in our study, the outcome of patients with intravascular embolization surgery was better.^[20]^ However, there was no significant difference in the mortality between cases undergoing craniotomy clipping and endovascular embolization surgeries in Hunt–Hess IV and V grade patients who required decompression surgery.^[21]^ The incidence rates of symptomatic cerebral vasospasm and hydrocephalus in patients with craniotomy clipping surgery were significantly higher than patients with intravascular embolization surgery.^[22]^ Moreover, some studies suggested that endovascular embolization was effective in treating subarachnoid hemorrhage and could alleviate immune, cognitive, and neurological dysfunction.^[23]^ However, some studies also suggest that the efficacy of endovascular embolization was comparable to that of craniotomy aneurysm clipping. A retrospective study including 583 patients with aneurysmal subarachnoid hemorrhage from multiple hospitals showed no significant difference in the GOS scores at discharge between patients with clipping and endovascular embolization surgeries. Moreover, multivariate analysis showed that clipping, clipping with cerebrospinal fluid drainage, endovascular embolization with hematoma clearance were all independently associated with good outcomes in patients with aneurysmal subarachnoid hemorrhage.^[24]^ In our study, endovascular embolization could induce a significant decrease of erythrocytes in cerebrospinal fluid on the 7th postoperative day. But craniotomy clipping could induce a decrease in cerebrospinal fluid pressure. Craniotomy clipping surgery possibly cause additional trauma to the brain, leading to an increase of erythrocytes in cerebrospinal fluid, but was possibly better than intravascular embolization in reducing cerebral pressure.

The release of cerebrospinal fluid through lumbar puncture and lumbar cistern drainage are important methods relieving blood stimulation to cerebral vessels. Patients who began early lumbar drainage at a rate of 5 mL/h within 72 hours after subarachnoid hemorrhage had fewer secondary infarcts upon discharge and reduced adverse outcome at 6 months.^[25]^ One meta-analysis results demonstrated that lumbar drainage of cerebrospinal fluid could reduce symptomatic vasospasm, cerebral infarction, subsequent intravascular treatment, and mortality. Reducing the stimulation of blood to cerebral vessels through lumbar puncture also proved that the poor outcome of patients was associated with the level of erythrocytes in subarachnoid space.^[26]^ By using hemoglobin, ferritin, and malondialdehyde as markers, Chen YH et al investigated whether continuous drainage could reduce erythrocytes in cerebrospinal fluid. On the 7th day after subarachnoid hemorrhage, levels of intrathecal hemoglobin, bilirubin, ferritin, and malondialdehyde were all reduced in patients with lumbar cistern drainage.^[27]^ Our study compared the effects of lumbar puncture and continuous lumbar cistern drainage on cerebrospinal fluid measurement results, and found no difference in the level of erythrocytes on the 7th postoperative day. Continuous cerebrospinal fluid drainage did not lead to a long lasting decrease of erythrocytes in cerebrospinal fluid. However, the level of leukocytes in cerebrospinal fluid was higher in patients with lumbar cistern drainage. We considered that continuous lumbar cistern drainage possibly leaded to an increase of leukocytes in cerebrospinal fluid and risk of intracranial infection.

Tranexamic acid is the main antifibrinolytic agent for aneurysmal subarachnoid hemorrhage. A randomized, prospective, multicenter study was conducted to evaluate the efficacy of short-term (not exceeding 72 hours) antifibrinolytic agent with tranexamic acid in preventing recurrent bleeding. There were 27 cases in patients without tranexamic acid treatment suffering from early rebleeding, of which 13 cases died. Six cases with tranexamic acid treatment experienced recurrence and 2 cases died. Tranexamic acid treatment resulted in a reduction of rebleeding rate from 10.8% to 2.4% and an 80% reduction in mortality rate. According to GOS results, the good outcome increased from 70.5% to 74.8%. However, there was no increased risk of ischemic manifestations or vascular spasms according to TCD measurements and clinical findings.^[28]^ In another study, the rebleeding rate of patients receiving short-term tranexamic acid treatment was similar to that of patients without tranexamic acid treatment, and the incidence of cerebral infarction was also similar. However, tranexamic acid treatment for 4 days did not reduce the incidence of rebleeding, but increased the incidence of cerebral infarction.^[29]^ Tranexamic acid treatment also had no effect on good clinical outcome or mortality, and was associated with increased hydrocephalus and seizures.^[30,31]^ We compared the effect of tranexamic acid treatment on the measurement results of cerebrospinal fluid, and found no advantage of tranexamic acid treatment in reducing erythrocytes, leukocytes, and pressure in cerebrospinal fluid. We also found no advantage of tranexamic acid treatment in the outcome of aSAH patients.

There were some limitations in our study. First, we were unable to obtain other cerebrospinal fluid results in a small number of patients due to retrospective nature of the study. Second, the cerebrospinal fluid measurement results on the 7th postoperative day were relatively few, which resulted in a bias in the comparisons. We need to further expand the sample size to study the impact of different cerebrospinal fluid release methods, and different surgery methods on the clinical outcome of patients.

## Acknowledgments

We are thankful to all the medical staff and statistician in the Neurointensive care unit, The First Affiliated Hospital of Yangtze University.

## Author contributions

**Conceptualization:** Jie Min, Yongfeng Zhao.

**Data curation:** Xian Wang.

**Formal analysis:** Jian Zhao.

**Methodology:** Xian Wang.

**Project administration:** Jie Min, Jian Zhao.

**Writing – original draft:** Jie Min.

**Writing – review & editing:** Yongfeng Zhao.
